# Short-Term Effect of a New Oral Sodium Hyaluronate Formulation on Knee Osteoarthritis: A Double-Blind, Randomized, Placebo-Controlled Clinical Trial

**DOI:** 10.3390/diseases8030026

**Published:** 2020-07-08

**Authors:** Arrigo F. G. Cicero, Nicolò Girolimetto, Crescenzio Bentivenga, Elisa Grandi, Federica Fogacci, Claudio Borghi

**Affiliations:** 1 Medical and Surgical Sciences Department, Sant’Orsola-Malpighi University Hospital, Alma Mater Studiorum University of Bologna, 40138 Bologna, Italy; crescenzio.bentivenga@aosp.bo.it (C.B.); elisa.grandi@unibo.it (E.G.); federicafogacci@gmail.com (F.F.); claudio.borghi@unibo.it (C.B.); 2 Department of Rheumatology, Azienda USL-IRCCS di Reggio Emilia, 42121 Reggio Emilia, Italy; nicolo.girolimetto@gmail.com

**Keywords:** hyaluronic acid, osteoarthritis, dietary supplements, symptoms, anti-pain use

## Abstract

Objective: the aim of this pilot study was to test the short-term effect of oral supplementation with a sodium hyaluronate with a large spectrum of molecular weights (FS-HA^®^) on the symptoms and functionality of knee osteoarthritis (OA). Methods: 60 subjects affected by clinical and/or radiological diagnosis of symptomatic knee OA were consecutively enrolled in a randomized, double blind, placebo-controlled, clinical trial. At randomization visit, at day 28 (visit 2), and day 56 (visit 3), the Western Ontario McMaster Universities Osteoarthritis Index (WOMAC), the Lequesne Functional Index (LFI) and the Visual Analogue Scale (VAS) for pain (VAS-p) were administered to the enrolled patients. Then, patients were asked how many times they used rescue medications (non-steroidal antinflammatory drugs–NSAIDs and/or anti-pain drugs) during the previous 4 weeks. Finally, the range of knee joint motion (ROM) was also instrumentally measured. Results: In FS-HA^®^ treated subjects, VAS-p, pain and total WOMAC score, LFI and ROM significantly improved compared to the baseline values (*p* < 0.05). At 60 days, the VAS-p and the pain WOMAC score were significantly lower after FS-HA^®^ treatment when compared with placebo as well (*p* < 0.05). The FS-HA^®^ treated subjects significantly reduced the weekly use of NSAIDs and/or antipain drugs when compared to the placebo-treated ones (*p* < 0.05). Conclusion: the oral supplementation with a FS-HA^®^ characterized by a large spectrum of molecular weight was associated with a short-term improvement in symptomatology and functionality of osteoarthritis-affected knees, and associated with a reduction in the use of NSAIDS and anti-pain drugs.

## 1. Introduction

Osteoarthritis (OA) is the most common joint disease and it is characterized by cartilage erosion, changes in subchondral bone, osteophyte formation and synovial inflammation [1,2]. More specifically, OA of the knee is a major cause of chronic pain and disability among the elderly population [3,4,5]. It is estimated that 250 million people worldwide are affected by knee OA [6]. Among adults 60 years of age or older, the prevalence of symptomatic knee OA is approximately 10% in men and 13% in women, dramatically increasing in older age. However, considering asymptomatic patients, the prevalence could be about one in three in adults [7].

Moreover, knee OA has negative impacts on socioeconomic factors (including impaired work performance and early retirement) [8,9] and on healthcare costs [10]. Data from the US Medicare Expenditure Panel Survey (1996–2005) showed that the medical cost for OA was $185.5 billion [11]. Although there is no treatment proved to prevent or reverse the structural changes that occur in OA, a proper treatment that alleviates symptoms [12] may improve the patient’s self-perceived quality of life [13,14].

In the degradation of articular cartilage, functional limitation and pain, underlies the quantitative and qualitative alteration of hyaluronic acid (HA), the main component of synovial fluid and cartilage, in a pathophysiological process influenced by a wide variety of risk factors, whose impact complicates the disease and radically reduces the quality of life of the patient [15]. In OA patients, HA is depolymerized and eliminated faster than in healthy subjects, due to chronic inflammation [16]. HA concentration is significantly decreased in patients with end-stage knee OA [17].

The therapeutic approaches for symptomatic knee OA included numerous pharmacologic and non-pharmacologic options [14,18]. Given the advanced age of patients with knee OA and their potential comorbidities, an ideal non-surgical treatment should reduce pain and improve functionality without systemic complication risks. Frequently utilized non-operative knee OA treatments include HA that is being administrated either by local injection or by oral administration [19,20].

HA used intra-articularly (IA-HA) in the treatment of OA is known to increase viscosity of the synovial fluid, facilitate gliding via layer formation on the cartilage and protect soft tissue from trauma by acting as a shock absorbent [16]; also soothes the pain and exerts an immunomodulatory effect on inflammatory cells [21]. HA has a delayed onset of action in comparison with intra-articular corticosteroids, but a longer-lasting benefit [22]. The progression of osteoarthritis leads to exposure of subchondral bone at a weight-bearing site at which the bone will then be subjected to abrasion and further damage.

Some studies have shown that oral administration of HA can lead to significant improvements in pain and function [23,24,25]. Recent research about HA bioavailability via oral intake, showed that its absorption seems to slightly occur with lower molecular weight (middleweight, 50–200 KDa). After 10 h from the intake of a single dose of 200 mg/day, a steady increase is observed for the three administered HAs (high weight >2000 KDa; middleweight 1000–1800 KDa; extra-low weight 50–200 KDa) contrary to negative control. Furthermore, intrinsic properties of polysaccharides such as anti-oxidant and anti-inflammatory activity, higher for lower molecular weights, are improved during enzymatic metabolism by the activation into smaller fragments, but are dramatically reduced when the chain length becomes too small [26].

For all these reasons, authors decided to use a different sodium hyaluronate, characterized by a large spectrum of molecular weight to be administered to patients suffering from OA symptoms with a daily dosage of 200 mg in a double-blind, randomized, placebo-controlled clinical trial.

The aim of this pilot study was to test the short-term effect of the oral supplementation with a FS-HA^®^ with a large spectrum of molecular weights on symptoms and functionality of knee OA.

## 2. Materials and Methods

### 2.1. Study Design

The proposed study is a pilot, randomized, double blind, placebo controlled, two parallel groups, clinical trial. Consecutive patients suffering of knee OA under treatment at the rheumatologic office of the Policlinico S. Orsola-Malpighi were screened for enrolment.

The study fully complied with the ethical guidelines of the Declaration of Helsinki and its protocol was approved by the Ethical Committee of the University of Bologna on March 13^th^, 2019 (Bologna, Italy; Code: OA_FS-HA2019). All participants signed a written informed consent to participate. The trial was carried out in line with the CONSORT statement.

Four visits have been scheduled, starting from preliminary screening, followed after 7–14 days by an enrollment visit, when patients were randomized to indistinguishable placebo or active treatment (FS-HA^®^, sodium hyaluronate, characterized by a large spectrum of molecular weight, ExceptionHYAL^®^ Jump, kindly furnished by Roelmi HPC, Origgio, Varese, Italy), 200 mg/day. Then the enrolled patients were visited after 4 (T28) and 8 (T56) weeks.

### 2.2. Criteria for Eligibility of Patients

The patient inclusion criteria were as follows: (1) aged between 30 and 85 years old; (2) body mass index (BMI) <35 kg/m^2^; (3) diagnosis of knee OA according to American College of Rheumatology criteria; (4) Kellgren–Lawrence radiological classification scale ≥2; (5) signed informed consent.

The exclusion criteria were the following: (1) infiltration or dietary supplementation with HA in the previous 6 months; (2) ongoing infiltration (with any drug) or surgery treatment on the affected knee; (3) previously experienced resistance to anti-inflammatory (both non-steroid and corticosteroid anti-inflammatory drugs) or/and anti-pain (paracetamol, codeine, tramadol) treatments, as an indirect marker of more severe disease; (4) known allergy/hypersensitivity to hyaluronic acid; (5) glucocorticoids in any usage form, 1 month before and during the investigation period; (6) each medical or surgical condition reducing the ability of the patient to comply with the study protocol.

### 2.3. Treatment

The randomization was performed at a ratio of 1:1 and the blocks were stratified by sex and age. An alphabetical code was assigned to each lot code (corresponding to treatment or placebo) impressed on the dose box. The study staff and the investigators, as well as all of the volunteers, were blinded to the group assignment. Codes were kept in a sealed envelope, which was not opened until the end of the trial. Dose boxes were mixed and a blinded dose box was assigned to each enrolled patient.

Treatment compliance was assessed by counting the number of pills returned at the time of specified clinic visits. At baseline, we weighed participants and gave them a bottle containing a supply of the study treatment for 60 days. Throughout the study, we instructed patients to take their first dose of new treatment product on the day after they were given it. All unused pills were retrieved for inventory. All treatment products were provided free of charge.

During all the follow up period, patients did not change the type of non-steroidal antinflammatory drugs (NSAIDs) and/or anti-pain drugs that they used until inclusion in the trial.

### 2.4. Outcome Assessment

Patients’ personal history, outcomes and physical examination were evaluated at baseline and at fourth and eighth and week post randomization visit (T28 and T56 respectively) by a clinical independent assessor blinded to treatment. The Western Ontario McMaster Universities Osteoarthritis Index (WOMAC), the Lequesne Functional Index (LFI) and the Visual Analogue Scale for pain (VAS-p) were administered to the enrolled patients. Clinical evaluation provided physical examination of the affected knee and a goniometer-based evaluation of the articular range of motion (ROM). Then, the patients were asked how many times they had used rescue medication (NSAIDs and/or anti-pain drugs) during the previous 4 weeks. Adherence, tolerability and acceptability of the tested treatments were also assessed at T28 and T56.

### 2.5. Statistical Analysis

Data were analyzed using intention to treat by means of the Statistical Package for Social Science (SPSS) version 21.0 (IBM Corporation, Armonk, NY, USA) for Windows. The normality distribution of the tested parameters was evaluated by the Kolmogorov–Smirnov test. The baseline characteristics of the population were described by the independent *t*-test and the χ^2^ test, followed by Fisher’s exact test for the categorical variables. Every continuous parameter was compared by repeated-measures analysis of variance (ANOVA). The intervention effects were adjusted for all of the considered potential confounders by the analysis of covariance (ANCOVA). ANOVA was performed in order to assess the significance within and between groups. The statistical significance of the independent effects of treatments on the other variables was determined by the use of the ANCOVA. A one-sample *t*-test was used to compare the values obtained before and after the treatment administration; 2-sample *t*-tests were used for between-group comparisons. Tukey’s correction was carried out for multiple comparisons.

All data were expressed as mean ± standard deviation (SD). Every test was two-tailed. *p*-values < 0.05 were always regarded as statistically significant.

## 3. Results

Sixty subjects were consecutively enrolled. The enrolled subjects age (FS-HA^®^ group: 56 ± 8 vs. placebo group: 57 ± 7 years), gender (FS-HA^®^ group: M:F = 11:14 vs. placebo group: M:F = 12:13), and BMI (FS-HA^®^ group: 26.1 ± 1.2 vs. placebo group: 25.9 ± 1.8 kg/m^2^) were matched between the two groups. All the enrolled subjects completed the trials without any clinically detectable adverse events registered in both groups of treatment. The compliance to the treatment was >90% in both groups of treatments.

Placebo-treated subjects did not experience any significant subjective nor objective improvement in their condition during the trial (*p* > 0.05). In FS-HA^®^ treated subjects, VAS-p, pain and total WOMAC score, LFI and ROM significantly improved compared to the baseline values (*p* always < 0.05). At 60 days, the VAS-p and the pain WOMAC score were significantly lower after FS-HA^®^ treatment when compared with placebo as well (*p* < 0.05) (Table 1).

**Figure 1:**
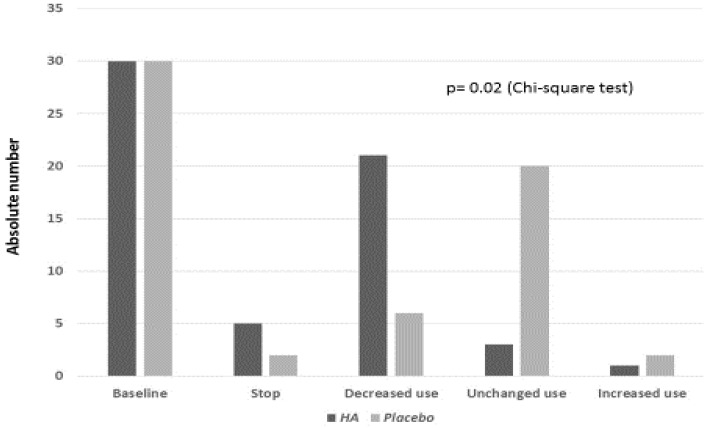
Modification of anti-pain and/or non-steroidal antinflammatory drugs (NSAIDs) weekly use pattern during the trial in the hyaluronic acid (HA) and placebo-treated subjects.

As regards the weekly pattern use of anti-pain and/or NSAIDs, it was significantly modified by the treatment with FS-HA^®^ (*p* < 0.05). In particular, the FS-HA^®^ treated subjects significantly reduced the weekly use of anti-pain and/or NSAIDs when compared to the placebo treated ones (*p* < 0.05) (Figure 1). The mean acceptability score was 9.3/10 in the FS-HA^®^ treated group and 8.9/10 in the placebo treated one, without significant difference between groups (*p* > 0.05).

## 4. Discussion

Simple analgesics, NSAIDs, tramadol, physical therapy agents, intra-articular injections (hyaluronic acid, steroid etc.) and surgical methods are commonly used in the treatment of OA [17,18,27]. However conventional pharmacological management for OA is often insufficient, and there is no consensus about the effects of each treatment on cartilage and synovial tissue. Previous research has found that HA, a glycosaminoglycan polymer chain, has shorter chain lengths, decreased molecular weight distribution, and lower concentration within osteoarthritic knees when compared to healthy knees [28,29].

Within the human body, HA is naturally synthesized in the plasma membrane of cells, thanks to HA synthases which produces unbranched single-chain polymers of disaccharide units of *N-*acetylamine and glucuronic acid. The synthesis results in high molecular weight HA which is released in the extracellular environment. For example, synovial fluid in healthy people contains HA with an average weight of about 7 × 106 Da, which if straightened would extend to more than 15 µm [30].

HA is physiologically degraded in an enzymatic way. In skin and joints, almost 20–30% of HA turnover occurs by local metabolism, and the rest is removed by lymphatic pathways.

The fact that HA is synthesized as a high molecular weight molecule, then degraded, allows a constant physiological balance of different HA fragments within the body. Therefore, if we imagine forming a picture of the synovial fluid of a healthy knee, it will be possible to find high molecular weights HA just synthesized, medium molecular weights HA in their degradation phase and low molecular weights HA which are going to be removed.

As all kinds of molecular weight HA can interact with cell receptors, giving different signals and stimulating different cell reactions, the balance of all these signals is the mechanism behind hyaluronic acid complex activity.

The primary role of synovial fluid is protective, by means of limiting axial forces on the articular surface and decreasing friction between joint surfaces. HA is entirely responsible for the elastoviscosity of synovial fluid. Because of its HA content, synovial fluid can behave as either a predominantly viscous fluid or an elastic fluid [31]. HA is also responsible for protecting the collagen fibrils and cells of articular surfaces, synovial tissue, capsules and ligaments from mechanical damage [32]. In osteoarthritis, the synovial fluid is more abundant and less viscous [33]. HA becomes depolymerized, its concentration and molecular weights are decreased, resulting in a decrease of elastoviscosity. These changes increase the susceptibility of cartilage to injury [34,35]. Osteoarthritic synovial fluid functions primarily as a viscous rather than elastic fluid through the entire range of joint movement, which reduces its protective effect on cartilaginous, fibrous, and cellular structures. As articular cartilage is progressively damaged, the net rate of proteoglycan synthesis ultimately falls, and the cartilage thins, resulting in a decrease in the load-bearing capacity [36].

Although commonly used in clinical practice, conflicting evidence for IA-HA efficacy has resulted in a lack of agreement among various guidelines regarding its use [37]. Indeed, there are no strict rules concerning the injection technique, age, radiographic staging of osteoarthritis, severity of symptoms, physical activity level, previous trauma or deformity; and therefore patient selection has not been clearly delineated [38]. The discrepancy between the beneficial effects of this procedure observed in clinical practice and guideline recommendations may result from study characteristics, such as inclusion criteria and the form of HA used. The inconsistent results within the current literature regarding the efficacy of IA-HA for the treatment of knee OA have been suggested to be due to intrinsic differences between individual HA products. Some IA-HA products are linear chain and some are mixtures of linear chain and chemically cross-linked HA. Currently, several HA formulations are approved for clinical use in Europe and the United States. These formulations differ in the origin of the HA and manufacturing process used, in their chemical–physical properties such as molecular weight and final concentration, joint space half-life, rheological properties, as well as their administration schedules and cost [39,40].

Different length chains behave differently: oligosaccharides have angio-genetic effects, low-weight polysaccharides have proliferative effects, while medium-high chains induce a quiescent response.

Some in vitro studies showed [20,41] that low and high molecular weight HA used together have a biological synergistic effect compared to the single molecular weight alone.

The average wholesale price for a six-month treatment of intra-articular HA injection greatly exceeds the cost of effective HA dietary supplements for the corresponding period. Currently, there have been no comparative reports regarding the cost-effectiveness of intra-articular injection versus dietary supplements. Moreover, so far, few randomized, double-blind, placebo-controlled trials have demonstrated the effectiveness of dietary HA in alleviating osteoarthritis symptoms, usually with short follow-ups and small sample size [25,42].

In our study, the oral supplementation with a FS-HA^®^ has demonstrated a short-term improvement in the symptomatology and functionality of osteoarthritis-affected knees. Our work represents the first insight into the use of a FS-HA^®^, expected to exert a higher affinity with cell-receptors, resulting in an improved efficacy profile in the short term, with regard to both symptoms, joint motility and function.

It is generally believed that it is difficult for the body to absorb a long-chain polysaccharide. Indeed, HA is not absorbed into the body as a high molecular weight polymer after ingestion. However, the body absorbs the high molecular weight polymer in the small intestine as 2–6 membered polysaccharides [43], decomposed by enteric bacteria. One proposed mechanism of action shows that ingested HA binds to Toll-like receptor-4 and promotes the expressions of interleukin-10 and cytokine signalling, which both lead to anti-inflammation of arthritis [44]. This report identified a signalling cascade in which receptors on intestinal epithelial cells are activated by oral HA which results in decreased pain. Oral HA binds to an intestinal receptor (Toll-like receptor-4; TLR-4). Cytokine array analysis showed that HA enhanced the production of interleukin-10 (IL-10), an anti-inflammatory cytokine. DNA array analysis of tissue from the large intestine showed that HA up-regulates suppressor of cytokine signalling 3 (SOCS3) expression and down-regulates pleiotrophin expression. These results suggest that the binding of HA to TLR-4 promotes IL-10 and SOCS3 expression and suppresses pleiotrophin expression leading to anti-inflammation of arthritis.

The observed improvement of symptoms and knee functionality mediated by the intake of FS-HA^®^ could have different long-term positive effect. Undoubtedly, it could improve the autonomy and self-perceived quality of life of patients. On the other hand, it could also prevent body weight increase and incident type 2 diabetes related to force sedentariety [45].

These results are in line with those observed with large doses of some well-studied cartilage-protecting agents, such as glucosamine and chondroitin, both exerting a certain degree of anti-inflammatory and anti-pain effect in OA patients [46].

Furthermore, in our study we registered a significant decrease in the use of NSAID and anti-pain drugs in FS-HA^®^ treated patients. This could have a relevant impact on the patients’ health. In fact, NSAIDs are the most prescribed agents for OA [47] and, as such, are expected to be associated with various side effects that include gastrointestinal, renal and cardiovascular implications [48,49]. This is even more so since their use is often incorrect and related with increased costs for the national health system. It is in fact reported that more than 30 million people worldwide use NSAIDs every day [50]; more than 111 million prescriptions for NSAIDs are dispensed in the United States of America (USA) annually, which accounts for approximately 60% of the USA over-the-counter analgesic market [51]. Moreover, recent studies have shown that the overall utilization of analgesics has increased considerably over the last decades, although there are substantial differences in trends toward utilization of particular analgesics among countries [52]. Beyond the risk of adverse events, NSAIDs and anti-pain drugs have no influence (if not negative) on the natural history of OA, simply temporarily reducing the perceived pain. By contrast, FS-HA^®^ use could be associated with a positive impact on joint health per se, even if no radiological evidence of long-term efficacy of condroprotective nutraceuticals is yet available.

The present study has several limitations, and the main of those is represented by the relatively short follow-up period. The follow-up of our analysis should be prolonged in order to reach solid conclusions, preferably with the support of a radiological evaluation of the lesion changes. Moreover, the current results should be considered as preliminary, and they need replication on a larger patient sample.

## 5. Conclusions

In this pilot study, oral supplementation with a FS-HA^®^ characterized by a large spectrum of molecular weights was associated with a short-term improvement in the symptomatology and functionality of osteoarthritis-affected knees, and associated with a reduction in the use of NSAIDS and anti-pain drugs. Further larger and long-term studies should be carried out to confirm these preliminary data.

## Figures and Tables

**Table 1:** Changes in VAS-p, WOMAC (and related subscales) indexes, Lequesne functional index (LFI) and knee extension ROM in the enrolled subjects during the trial.

	FS-HA (*n*: 30)			Placebo (*n*: 30)		
	T0	T28	T56	T0	T28	T56
VAS-p (mean ± SD)	6.7 ± 1.0	5.5 * ± 0.9	4.1 *^,^° ± 0.6	6.4 ± 1.1	5.9 ± 1.2	6.0 ± 1.3
Pain WOMAC (mean ± SD)	9.6 ± 1.2	9.0 * ± 1.2	8.8 *^,^° ± 0.9	9.3 ± 1.4	9.1 ± 1.2	9.3 ± 1.1
Function WOMAC (mean ± SD)	22.8 ± 2.4	22.1 ± 2.5	20.3 ± 1.9 *	23.1 ± 2.7	22.9 ± 2.8	22.7 ± 2.1
Total WOMAC (mean ± SD)	40.3 ± 3.8	36.8 * ± 4.3	33.9 *^,^° ± 4.1	40.5 ± 3.8	39.3 ± 4.1	38.9 ± 4.4
Lequesne Functional Index (mean ± SD)	6.5 ± 0.9	6.3 ± 1.0	6.1 * ± 1.1	6.7 ± 1.1	6.5 ± 1.0	6.5 ± 1.2
Extension ROM (mean ± SD)	86 ± 11	88 ± 12	91 ± 15 *	85 ± 13	84 ± 11	82 ± 13

Legend: ROM: range of motion; T0: baseline; T28: 4-week follow up; T56: 8-week follow up; VAS-p: visual analog scale for pain; WOMAC: Western Ontario McMaster Universities Osteoarthritis Index; * *p* < 0.05 vs. baseline; ° *p* < 0.05 vs. placebo.
